# Clinical applications of electrical stimulation for peripheral nerve injury: a systematic review

**DOI:** 10.3389/fnins.2023.1162851

**Published:** 2023-08-03

**Authors:** Meredith C. Costello, Emily L. Errante, Taylor Smartz, Wilson Z. Ray, Allan D. Levi, Stephen Shelby Burks

**Affiliations:** ^1^Department of Neurological Surgery, University of Miami Miller School of Medicine, Miami, FL, United States; ^2^The Miami Project to Cure Paralysis, Miami, FL, United States; ^3^Department of Neurological Surgery, Washington University School of Medicine, St. Louis, MO, United States

**Keywords:** peripheral nerve injury, electrical stimulation, clinical application, recovery, reinnervation

## Abstract

**Introduction:**

Peripheral nerve injuries are common neurologic injuries that are challenging to treat with current therapies. Electrical stimulation has been shown to accelerate reinnervation and enhance functional recovery. This study aims to review the literature on clinical application of electrical stimulation for peripheral nerve injury.

**Methods:**

PubMed and Embase were sourced from 1995 to August 2022. Selection was based on predetermined inclusion/exclusion criteria. Eight hundred and thirty-five articles were screened with seven being included in this review.

**Results:**

Two hundred and twenty-nine patients with peripheral nerve injuries were represented. Six of the studies were randomized controlled trials. A variety of nerve injuries were represented with all being in the upper extremity and supraclavicular region. Electrical stimulation protocols and evaluation varied. Electrodes were implanted in four studies with one also implanting the stimulator. Length of stimulation per session was either 20 mins or 1 h. Median stimulation frequency was 20 Hz. Stimulation intensity varied from 3 to 30V; pulse width ranged from 0.1 to 1.007 ms. Three protocols were conducted immediately after surgery. Patients were followed for an average of 13.5 months and were evaluated using electrophysiology and combinations of motor, sensory, and functional criteria.

**Discussion:**

Patients who received electrical stimulation consistently demonstrated better recovery compared to their respective controls. Electrical stimulation for peripheral nerve injury is a novel treatment that has not been well-studied in humans. Our review illustrates the potential benefit in implementing this approach into everyday practice. Future research should aim to optimize protocol for clinical use.

## Introduction

Peripheral nerve injuries are common neurologic injures and are challenging to treat adequately with current surgical techniques. Although peripheral nerve axons have the potential to regenerate, functional recovery after nerves injuries remains challenging particularly when longer distances are required for motor and sensory reinnervation. One method of interest to aid in regeneration is the use of electrical stimulation (ES), which has been shown to both accelerate reinnervation and enhance functional recovery (Gordon, 2016; Chu et al., 2022). Prior research has demonstrated that ES may address the complex pathophysiology involved in the inhibition of synaptic stripping and the excessive excitability of the dorsal root ganglion, while alleviating neuropathic pain, improving neurologic function, and accelerating nerve regeneration (Chu et al., 2022). Investigation of ES for peripheral nerve injury began in the mid-twentieth century when Hoffman (1952) demonstrated accelerated axon sprouting at various frequencies. Following this initial study, ES has been shown to be most promising when applied immediately following nerve injury with brief stimulation for 1 h at 20 Hz (Al-Majed et al., 2000a; Brushart et al., 2005; Ahlborn et al., 2007; Lal et al., 2008; Sharma et al., 2010; Singh et al., 2012; Witzel et al., 2016). In addition to peripheral nerve regeneration, ES has also been investigated for treatment of a variety of neurologic injuries or chronic conditions, including spinal injuries, traumatic brain injuries, and neuropathic pain (Peri et al., 2001; Johnson and Burchiel, 2004; Cheing and Luk, 2005; Jarrett et al., 2005; Shields and Dudley-Javoroski, 2006; Oosterhof et al., 2008; Deer et al., 2010; Lee et al., 2012; Lairamore et al., 2014; Oo, 2014; Chen et al., 2015; Gall et al., 2016; Wu et al., 2017; Redshaw et al., 2018; Stampas et al., 2019; Liechti et al., 2020; Johnson et al., 2021; Kamboonlert et al., 2021). Although a relatively new therapy, ES has been demonstrated to be a safe and effective adjunctive therapy with established treatments including surgical repair, pharmacologic treatment, and cell-based therapies (Chu et al., 2022).

The preclinical data supporting electrical stimulation following peripheral nerve injury is robust and shows an overwhelming support for its use. After peripheral nerve injury, nerve growth factor (NGF) production in Schwann cells declines, limiting neural repair (Huang et al., 2010a). However, animal studies have shown that ES may be able to counteract this through stimulating dorsal root ganglions and Schwann cells to increase production of cyclic adenosine monophosphate (cAMP) and NGF respectively (Udina et al., 2008; Huang et al., 2010b). Because cAMP counteracts myelin inhibition and allows axon regeneration, increased levels after ES is proposed to enhance neurite outgrowth and extension (Aglah et al., 2008). ES stimulates peripheral nerve regeneration-associated genes within the cell body, namely Talpha1-tubulin and growth associated protein 43 (GAP-43) expression, while also reducing medium-molecular-weight neurofilament mRNA (Al-Majed et al., 2004; Chu et al., 2022). This change in the neurofilament/tubulin expression ratio is thought to allow more tubulin to be transported at a faster rate there by accelerating elongation (Al-Majed et al., 2004). Brief electrical stimulation also stimulates brain derived neurotrophic factor (BDNF) and its receptor, tyrosine receptor kinase B (trkB), expression in regenerating motoneurons which is believed to promote axonal regeneration through an autocrine and/or paracrine function (Al-Majed et al., 2000b; English et al., 2007).

Further, it has been noted in the literature that ES proximal to the site of peripheral nerve injury leads to therapeutic benefits (Chu et al., 2022). In one study, researchers examined continuous 20 Hz ES applied at variable durations (1 h to 2 weeks) in a rat, femoral nerve transection model to assess motor axonal regeneration (Al-Majed et al., 2000a). Although it was established that the processes associated with preferential motor reinnervation take 10 weeks to occur, ES dramatically reduced this period to 3 weeks, demonstrating its ability to substantially augment recovery (Al-Majed et al., 2000a). English and colleagues have also shown convincing data demonstrating the benefits of ES when applied to a sciatic injury model (English et al., 2007). In their study, they were able to show accelerated axonal growth in common fibular nerves that had been subjected to ES proximally compared to those that had received sham ES (English et al., 2007). Taken together, these studies and several others on the subject demonstrate the clear efficacy of ES in animal models with recovery after injury.

While there is extremely promising data on ES in animal models (Al-Majed et al., 2000b; Geremia et al., 2007; Gordon et al., 2009; Xu et al., 2014; Elzinga et al., 2015; Willand et al., 2016; Shapira et al., 2019), the applicability of this approach to humans is still under investigation. Relatively few clinical trials have been completed in human populations leaving optimal protocol, including electrode arrangements, and application methods unknown. Regarding the frequency and duration of ES, some hypothesize that any stimulation beyond the initial aforementioned 1-h brief stimulation may be dose dependent (Javeed et al., 2021).

For ES to be implemented in a clinical setting, a multifaceted approach targeting the different aspects of regeneration is essential. ES in conjunction with other rehabilitation methods, such as exercise, must be investigated before confidently transitioning to clinical application (Javeed et al., 2021). In this systematic review, we examine the current literature surrounding clinical application of electrical stimulation for peripheral nerve injuries to consolidate current findings and provide next steps for implementation into everyday practice.

## Methods

Following PRISMA guidelines, PubMed and Embase were sourced for clinical trials between 1995 and August 2022 (Page et al., 2021). The search terms (peripheral nerve stimulation) AND (nerve injury) were used to retrieve articles of relevance. Inclusion criteria included clinical application on human subjects, sustainment of a peripheral nerve injury, and specification of nerve injured. Additionally, included studies must have outlined stimulation protocol and evaluation criteria used during follow-up. Case reports, studies involving central nervous system injuries, and studies for neuropathic pain control were excluded. Two reviewers independently screened articles for inclusion to reduce the risk of bias. One reviewer extracted data which was later verified by a second reviewer to ensure accuracy and also reduce bias. Given the small number of relevant studies, a meta-analysis was foregone (Cheung and Vijayakumar, 2016). Study type, size, design, and results were collected from each of the included studies. Additionally, stimulation protocols and evaluation criteria were also collected.

## Results

**Figure 1:**
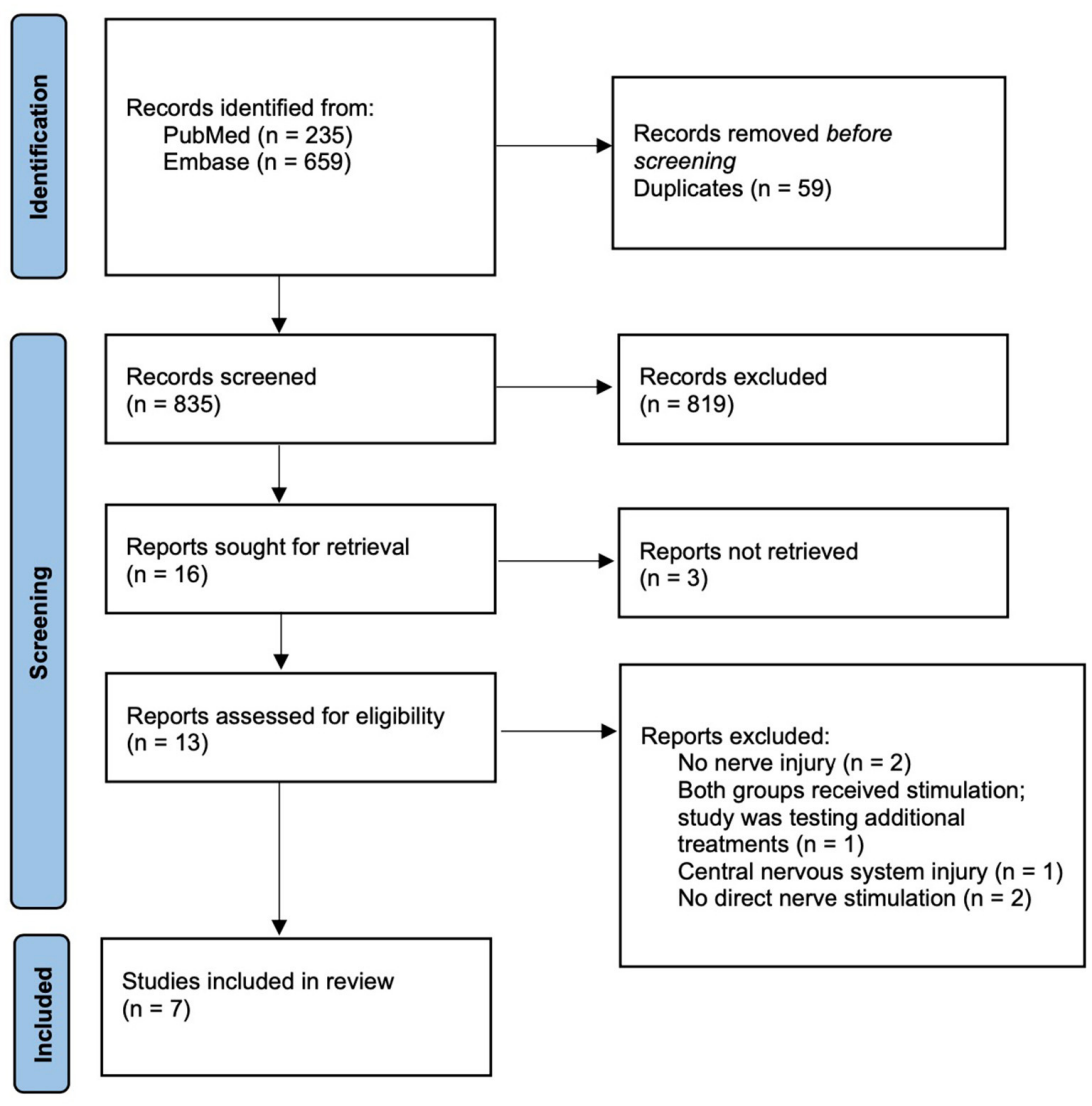
PRISMA diagram outlining study selection process (Page et al., 2021).

Eight hundred thirty-five articles were initially generated and were screened using predetermined inclusion and exclusion criteria. A total of seven studies ultimately met the review criteria and were included. Figure 1 outlines the process of study selection. A significant number of sourced studies were excluded from analysis; however, the majority were excluded because they investigated ES for purposes outside the scope of this study. Between the seven included studies, a total of 229 patients with peripheral nerve injuries were represented. All included studies were a variation of a randomized controlled trial except for the study by Williams (1996) which was a pilot trial. A variety of nerve injuries were studied with all being in the upper extremity or supraclavicular region. Four studies included injuries from chronic compression of the median or ulnar nerves, one study involved injury following surgical repair for digital transection, and one study investigated injury after surgical retraction and devascularization of the spinal accessory nerve. Williams (1996) included a variety of upper extremity nerve injuries and did not report mechanism of injury. An overview of the clinical trials is outlined Table 1 (Williams, 1996; Naeser et al., 2002; Gordon et al., 2010; Koca et al., 2014; Wong et al., 2015; Barber et al., 2018; Power et al., 2020).

**Table 1:** Overview of clinical trials.

**Author & Year**	**Study type**	**Size**	**Nerve injured**	**Type of injury**	**Treatment**	**Results**	**Conclusion**
Power et al. (2020)	Double blind RCT	*n* = 31 patients with CuTS	Ulnar	Chronic compression	Surgical treatment^a^ + PES: *n* = 20 Surgical treatment^a^ alone: *n* = 11	After three years of follow-up, stimulated patients had more than double the number of motor units, clinically important increases in grip strength and key pinch strength, and higher McGowan-Goldberg grades when compared to controls.	PES enhances reinnervation of muscles and improves functional recovery beyond what is seen with surgery alone.
Barber et al. (2018)	Double blind RCT	*n* = 54 patients undergoing oncologic neck dissection	Spinal accessory	Surgical retraction and devascularization	No stimulation: *n* = 27 ES: *n* = 27	Patients who received ES had significantly higher CMS scores in the intent-to-treat analysis. NDII and CMAP values were improved in the per-protocol analysis.	ES may enhance regeneration in more types of peripheral nerve injuries than previously considered.
Wong et al. (2015)	Double blind RCT	*n* = 36 patients from plastic surgery hand clinics at the University of Alberta	Digital	Transection	Surgical repair^b^ + ES: *n* = 18 Surgical repair^b^ alone: *n* = 18	Patients who received ES recovered near-normal sensation at 5-6 months, but control patients did not. Although there was a greater functional improvement in the ES group, there was no statistically significant difference when compared to control.	Delivery of ES for nerve laceration and repair is feasible. ES can be used for both distal and proximal nerve injuries where there is both motor and sensory involvement.
Koca et al. (2014)	Single blind RCT	*n* = 63 patients with CTS	Median	Chronic compression	Splint: *n* = 22 TENS: *n* = 20 IFC: *n* = 21	IFC therapy provided a significantly greater improvement in VAS, mMDL, and mSNCV values than splint therapy. However, there was no significant difference in improvement between TENS and splint group. IFC therapy provided a significantly greater improvement in VAS, symptom severity, functionality, mMDL and mSNCV values than TENS therapy.	IFC may provide an increase in local circulation and a decrease in interstitial edema through its stimulatory and pumping effects on local muscles. This may contribute to improved transmission along the median nerve.
Gordon et al. (2010)	RCT	*n* = 21 patients with CTS	Median	Chronic compression	CTRS + ES: *n* = 11 CTRS alone: *n* = 10	When compared to control group, patients who received ES had significant improvement in axonal regeneration with increases in MUNE. They also had significantly improved acceleration in terminal motor latency and an earlier increase in sensory nerve conduction values.	Brief low frequency electrical stimulation accelerates axonal regeneration to promote complete muscle reinnervation in humans.
Naeser et al. (2002)	Double blind crossover RCT	*n* = 11 patients with CTS who failed standard medical or surgical treatment	Median	Chronic compression	Laser therapy + TENS: *n* = 11	After receiving the treatment series, significant decreases in MPQ scores, median nerve sensory latency, and Phalen and Tinel signs were observed. Patients were able to return to work and were stable for 1–3 years.	LLLT and microamps TENS may be an effective conservative treatment for patients with CTS, especially when applied at earlier stages.
Williams (1996)	Pilot study	*n* = 13 patients with surgically repaired upper extremity nerve injuries	Median (*n* = 5) Ulnar (*n* = 2) Combined Median/Ulnar (*n* = 2) Radial (*n* = 4)	NR	Continuous ES using implanted device: *n* = 13	All patients had satisfactory to excellent revovery based on clinical examination, EMG, and functional analysis. Motor recovery was generally better than sensory recovery. The best results were seen in patients with more distal nerve lesions.	Continuous stimulation using an implantable system is effective, tolerated well by patients, and eliminates concerns of patient compliance.

RCT, randomized control trial; CuTS, cubital tunnel syndrome; PES, postsurgical electrical stimulation; ES, electrical stimulation; CMS, Constant-Murley score; NDII, Neck Dissection Impairment index; CMAP, compound muscle action potential; CTS, carpal tunnel syndrome; IFC, interferential current; TENS, transcutaneous electrical nerve stimulation; VAS, visual analog scale; mMDL, median nerve motor distal latency; mSNCV, median sensory nerve conduction velocity; MUNE, motor unit number estimation; MPQ, McGill Pain Questionnaire; LLLT, low-level laser therapy; NR, not reported; EMG, electromyography.

a Surgical treatment consisted of *in situ* decompression or submuscular transposition.

b Surgical repair consisted of nerve end debridement and standard tension-free epineurial repair.

Electrical stimulation protocols and evaluation criteria were compared from all studies. Although protocol specifics varied, similar components were demonstrated throughout. The majority of protocols (*n* = 5) used bipolar stimulation. Electrodes were implanted in four studies with one also implanting the electrical stimulator. Four studies included only one session of stimulation and two studies explored the use of multiple stimulation sessions over the course of a few weeks. Williams (1996) explored the use of continuous stimulation to denervated muscle until nerve regeneration was determined to be complete upon assessment. The length of stimulation per session was either 20 mins or 1 h (excluding). The median stimulation frequency for six of the studies was 20 Hz (range 20–130 Hz). Naeser et al. (2002) explored the use of multiple frequencies during their protocol. Stimulation intensity varied from 3 to 30 V with most studies reporting a range used. Stimulation pulse width was also reported and ranged from 0.1 to 1.007 ms. Three protocols were conducted immediately after surgery, either in the operating room or in the post-anesthesia care unit. Three were conducted in a laboratory setting with Gordon et al. (2010) utilizing local anesthesia (1% lidocaine) at the site of surgical incision (Williams, 1996). While lidocaine is a voltage-gated sodium channel blocker, which could prevent action potential propagation, when injected into the epidermis it should have very little effect on ES. Given that Williams (1996) implemented continuous stimulation for months at a time using implantable stimulators, patients were able to complete their daily activities while receiving electrical stimulation. Differences in electrode placement can necessitate adjustment to stimulation parameters. Tables 1–3 outline the differences in the included studies that require variations in protocol. Across all studies, patients were followed for an average of 13.5 months and were evaluated using electrophysiology as well as combinations of motor, sensory, and functional criteria. Tables 2, 3 detail the electrical stimulation protocols and evaluation components for each study (Williams, 1996; Naeser et al., 2002; Gordon et al., 2010; Koca et al., 2014; Wong et al., 2015; Barber et al., 2018; Power et al., 2020).

**Table 2:** Electrical stimulation protocol.

**Study**	**Stimulation mode**	**Placement of electrode(s)**	**Stimulator used**	**Duration**	**Frequency**	**Intensity**	**Pulse width**	**Anesthesia**	**Procedure setting**
Power et al. (2020)	Bipolar	External	Grass Technologies SD9 Stimulator	1 h	20 Hz	< 30 V	0.1 ms	General	PACU
Barber et al. (2018)	Monopolar	Implanted^a^	Grass Technologies SD9 Stimulator	1 h	20 Hz	3–5 V	0.1 ms	General	OR
Wong et al. (2015)	Bipolar	Implanted	Grass Technologies SD9 Stimulator	1 h	20 Hz	< 30 V	0.1–0.4 ms	General	OR
Koca et al. (2014)	Bipolar	External	Chattanooga Intelect Legend XT 2 Channel Combination System	15 sessions (5/week) of 20 mins	100 Hz (TENS)	NR	80 ms	None	Lab
Gordon et al. (2010)	Bipolar	External	Grass Technologies SD9 Stimulator	1	20 Hz	4–6 V	0.1–0.8 ms	Local^b^	Lab
Naeser et al. (2002)	Monopolar	External	MicroStim Inc. Model 100 stimulator	9–12 treatments of 20 mins	292 Hz for 2 mins then 0.3 Hz for 18 mins	NR	NR	None	Lab
Williams (1996)	Bipolar	Implanted^c^	Medtronic Itrel 7424 stimulator	Patient specific (range= 123–317 days)	130 Hz	2–10.5 V	1.007 ms	None	NA^d^

Hz, hertz; V, volts; ms, milliseconds; PACU, Post anesthesia care unit; OR, operating room; mins, minutes; NR, not reported; TENS, transcutaneous electrical stimulation; NA, not applicable.

a Electrode cuff was placed around spinal accessory nerve.

b Local anesthesia (1% lidocaine) was utilized only at the site of surgical incision prior to decompressive surgery.

c Stimulated muscle directly using an implantable stimulator to assess nerve regeneration.

d Patients were stimulated continuously day and night until the regenerating nerve reached the involved muscle bellies.

**Table 3:** Follow-up and evaluation.

**Author & year**	**Follow-up time**	**Electrophysiology**	**Motor evaluation**	**Sensory evaluation**	**Functional evaluation**
Power et al. (2020)	36 mo	NCS MUNE	Grip strength, pinch strength, and McGowan-Goldberg grade	McGowan-Goldberg grade	NA
Barber et al. (2018)	12 mo	NCS CMAP	NA	NA	CMS NDII
Wong et al. (2015)	6 mo	NA	NA	Temperature, spatial discrimination, and pressure threshold	DASH questionnaire
Koca et al. (2014)	6 weeks	NA	mMDL	mSNCV	BCTQ, VAS, symptom severity scale
Gordon et al. (2010)	12 mo	NCS MUNE	Purdue pegboard test	SWM	Levine's questionnaire
Naeser et al. (2002)	NR	NA	Motor latency measure	Sensory latency measure	MPQ score, Tinel and Phalen signs
Williams (1996)	Patient specific^a^	EMG	Muscle grading, grip strength, pinch strength, muscle size, and range of motion	NA	NA

mo, month; NCS, nerve conduction study; MUNE, motor unit number estimation; NA, not applicable; CMAP, compound muscle action potential; CMS, Constant Murley score; NDII, Neck dissection impairment index; DASH, disabilities of arm; shoulder; and hand; mMDL, median nerve motor distal latency; mSNCV, median sensory nerve conduction velocity; VAS, visual analog scale; BCTQ, Boston carpal tunnel syndrome questionnaire; SWM, Semmes Weinstein Monofilaments; MPQ, McGill Pain Questionnaire; EMG, electromyography.

a Patients were seen and examined every 3 months until reinnervation was complete.

Patients who received electrical stimulation consistently demonstrated better recovery when compared to their respective controls. Power et al. (2020) found that after three years of follow-up, electrically stimulated patients had more than double the number of motor units and higher McGown-Goldberg grades than the control group. Barber et al. (2018) demonstrated significantly higher functional recovery among patients receiving electrical stimulation. Similarly, Wong et al. (2015) also demonstrated improved functional recovery in addition to a greater sensory recovery than the respective control group. Like Gordon et al. (2010) also demonstrated a significant increase in motor units as well as improved axonal regeneration, terminal motor latency, and earlier sensory nerve conduction values. Significant improvements in sensation and symptoms of nerve injury were also demonstrated by Naeser et al. (2002). Although Williams (1996) did not have a control for comparison, all patients demonstrated satisfactory to excellent recovery based on evaluation components.

## Discussion

Electrical stimulation for peripheral nerve injury is a relatively new approach to treatment that has not been well studied in human populations. Our review highlights the potential value and utility of adopting this treatment modality into clinical practice. Each of the studies included in this review conclude that ES may aid in nerve recovery and function. Patients receiving ES outperformed their control counterparts in motor, sensory, and/or functional recovery. To our knowledge, this is the first comprehensive systematic review of clinical trials investigating electrical stimulation for peripheral nerve injuries. Despite common themes being present throughout the ES protocols highlighted in this review, variations in type of stimulation, duration, frequency, intensity, pulse width, along with sex and timing of surgical nerve repair are difficult to account for given the relative scarcity of literature. Although an ample amount of research has been done using animal models, human clinical trials are needed to further elucidate their impacts on patient outcomes. Further investigation into the applicability, usefulness, and feasibility of this approach in human populations is needed.

While all studies were grouped together for the purposes of this review, there are differences worth mentioning among the studies across various factors. While one of the seven included studies was a transection injury, four of the others were chronic compression injuries. The remaining two studies included one study that involved a surgical retraction and devascularization and another study that did not report the type of injury. Overall, all the included studies examined injured nerves from the upper extremity; however, one study involved the spinal accessory nerve, one involved the digital nerves, one involved the ulnar nerve, three examined the median nerve, and the final study examined a combination of median and ulnar nerves. Although all included studies utilized ES, the protocol for ES treatment was different across the seven studies. For instance, two studies that involved surgical intervention utilized ES in two different ways, with one of the studies using ES during the repair and the other study using ES after the surgery had taken place. Interestingly, even though these and other differences were present among the seven studies, outcomes were generally the same. Specifically, those that were treated with ES showed improvement compared to those that were treated without ES, helping to demonstrate the efficacy of ES as a potential treatment after peripheral nerve injury (for overview of studies, see Table 1).

Although there are few studies pertaining to ES for peripheral nerve injury, this treatment modality has the potential to substantially change the management of these injuries. There has been no other pharmacological or interventional option that has provided the robust improvement in clinical outcomes like ES. As demonstrated by this review, ES has been shown to aid in functional recovery of peripheral nerve injuries. Significantly, this functional recovery was seen across a variety of peripheral nerves located in the upper extremities with similar success; however, it would be pertinent to assess the ability of ES to aid in functional recovery after lower extremity injury. The next step in advancing this treatment is further application in the clinical setting to possibly generate more data supporting its use in injuries beyond the scope of this review. By utilizing the work already put forward (Viv et al., 2008; Gordon et al., 2010; Wong et al., 2015; Gordon and English, 2016; Barber et al., 2018; Power et al., 2020) as a guide, future research into clinical outcomes may change current guidelines for addressing these types of injuries.

In this review, we summarized the available literature surrounding clinical applications of electrical stimulation for peripheral nerve injuries. Using the seven included studies, we were able to review the implementation of this treatment on a relatively large patient population. Our review also included studies that investigated implementation for multiple peripheral nerves and mechanisms of injury. The studies included had adequate follow-up time, allowing evaluation of nerve recovery over an average of more than one year. This, coupled with the ability to assess nerve injuries using multiple methods, provided a thorough and adequate assessment of the utility of this approach.

Use of electrical stimulation for the treatment of peripheral nerve injures is a relatively new approach to care that has not yet been well studied. However, despite the scarcity of literature detailing its use, initial reports appear promising and highlight the benefit of this approach to potentially enhance and accelerate functional recovery. Importantly, based on these studies, the advantages and disadvantages of both bipolar and monopolar ES should be considered prior to their use. Studies have shown that there are unique differences between the two electrode configurations, including differences in threshold amplitude, relative gain, and selectivity (Grandjean and Mortimer, 1986). Specifically, although the threshold amplitude is lower with monopolar configurations, the relative gain is decreased in bipolar configurations (Grandjean and Mortimer, 1986). While preclinical and clinical models have utilized monopolar ES with much success, others have argued that bipolar ES is preferred as it allows for the cathode and anode to be placed directly on the nerve, thereby leading to better focused treatment (López, 2010). Clinicians should consider both options of stimulation prior to selection. Further, before wide-scale implementation into clinical settings, future research is needed and should be aimed to resolve such translational concerns.

### Limitations

This study is not without limitations. Given the relatively small number of relevant studies, a meta-analysis was foregone (Cheung and Vijayakumar, 2016; Muka et al., 2020). Without this analysis, definitive conclusions could not be drawn regarding clinical trial variations. However, systematic reviews, even those not including a meta-analysis, are vital for linking research to practice (Cook et al., 1997; Mulrow et al., 1997). Additionally, many of the measures used, such as MUNE, are only currently available in the laboratory setting limiting their use in the clinical environment. Cost effectiveness and technical limitations also impede ES adaptation.

## Conclusions

Peripheral nerve injuries are common, but complete recovery is hard to achieve with current practices. Electrical stimulation for nerve injuries is a relatively new therapy that shows promising outcomes when used in adjunct with known treatment options. This review demonstrates that patients who received electrical stimulation consistently experienced better recovery when compared to their respective controls. Guidelines for use of electrical stimulation for peripheral nerve injury have not been established. Future work should focus on determining how to best incorporate this treatment for a variety of peripheral nerve injuries into the clinical environment.

## Data availability statement

The original contributions presented in the study are included in the article/supplementary material, further inquiries can be directed to the corresponding author.

## Author contributions

MC, EE, and SB contributed to idea conception and study design. MC organized the database. MC wrote the manuscript with assistance of TS and EE. SB, AL, and WR provided edits and assisted in manuscript revision. All authors have read and approved the submitted version.
